# User Experience Evaluation of a Spinal Surgery Robot: Workload, Usability, and Satisfaction Study

**DOI:** 10.2196/54425

**Published:** 2024-04-01

**Authors:** Hyeonkyeong Choi, Seunghee Kim, Wonseuk Jang

**Affiliations:** 1 Department of Medical Device Engineering and Management Yonsei University College of Medicine Seoul Republic of Korea; 2 Medical Device Usability Research Center Gangnam Severance Hospital Yonsei University College of Medicine Seoul Republic of Korea

**Keywords:** robot spine surgery, usability, satisfaction, System Usability Scale, surgical navigation systems, robotics, surgery, neurosurgery

## Abstract

**Background:**

Robotic spine surgery has continued to evolve since its US Food and Drug Administration approval in 2004, with products now including real-time video guidance and navigation during surgery. As the market for robotic surgical devices evolves, it is important to consider usability factors.

**Objective:**

The primary objective of this study was to determine the user experience of a surgical-assistive robotic device. The secondary objective was to evaluate workload, usability, the After-Scenario Questionnaire (ASQ), and the System Usability Scale (SUS). In addition, this study compares the workload, usability, and satisfaction survey of the device among different occupational groups using the device.

**Methods:**

Doctors (n=15) and nurses (n=15), the intended users of the surgical assistant robot, participated in the usability evaluation. Participants performed essential scenarios for the surgical assistant robot and provided scenario-specific satisfaction (ASQ), workload (NASA Task Load Index), and usability (SUS) scores.

**Results:**

Both doctors and nurses had task success rates of 85% or higher for each scenario. ASQ results showed that both doctors and nurses were least satisfied with ease of completing the task of registration (group 1: mean 4.73, SD 1.57 and group 2: mean 4.47, SD 1.8), amount of time it took (group 1: mean 4.47, SD 1.63 and group 2: mean 4.40, SD 2.09), and support information satisfaction (group 1: mean 5.13, SD 1.50 and group 2: mean 5.13, SD 1.89). All participants had low workloads, and the overall Task Load Index score had a *P* value of .77, which is greater than .05. The SUS results showed that the overall usability mean for doctors was 64.17 (SD 16.52) and the mean for nurses was 61.67 (SD 19.18), with a *P* value of .84, which is greater than .05, indicating no difference between the 2 groups.

**Conclusions:**

In this study, doctors and nurses evaluated the interaction of the device in a simulated environment, the operating room. By evaluating the use experience and usability of the device with real intended users, we can develop a more effective and convenient user interface.

## Introduction

### Background

Spine surgery is used to treat degenerative diseases and deformities of the spine, with 45 million surgeries performed annually in the United States [1]. The use of robotic-assisted navigation is increasing as the number of patients undergoing lumbar spinal fixation increases [2]. Spine surgery typically involves 7 people in the operating room, with an operator surgeon, a surgical first assistant (who may be a doctor or physician assistant nurse, depending on operating room staffing), and scrub nurse in the sterile area and a circulating nurse and radiologist in the nonsterile area. Nonoperative personnel include an anesthesiologist and an anesthesiologist assistant. The use of robotics in spine surgery is usually reserved for difficult anatomical areas where it is difficult to fix screws blindly. Spinal fusion surgery is the insertion and fixation of pedicle screws into the vertebrae to eliminate pain by preventing movement between vertebrae [3]. It is also used for quick insertion in severe scoliosis, collapsed vertebrae, or long-level fusion in patients with difficult anatomy, usually at the iliac screw, C1, C2, C7, T1, and T2, or for other reasons. This is usually used for kyphosis and scoliosis correction.

### Robot Spine Surgery

**Figure 1:**
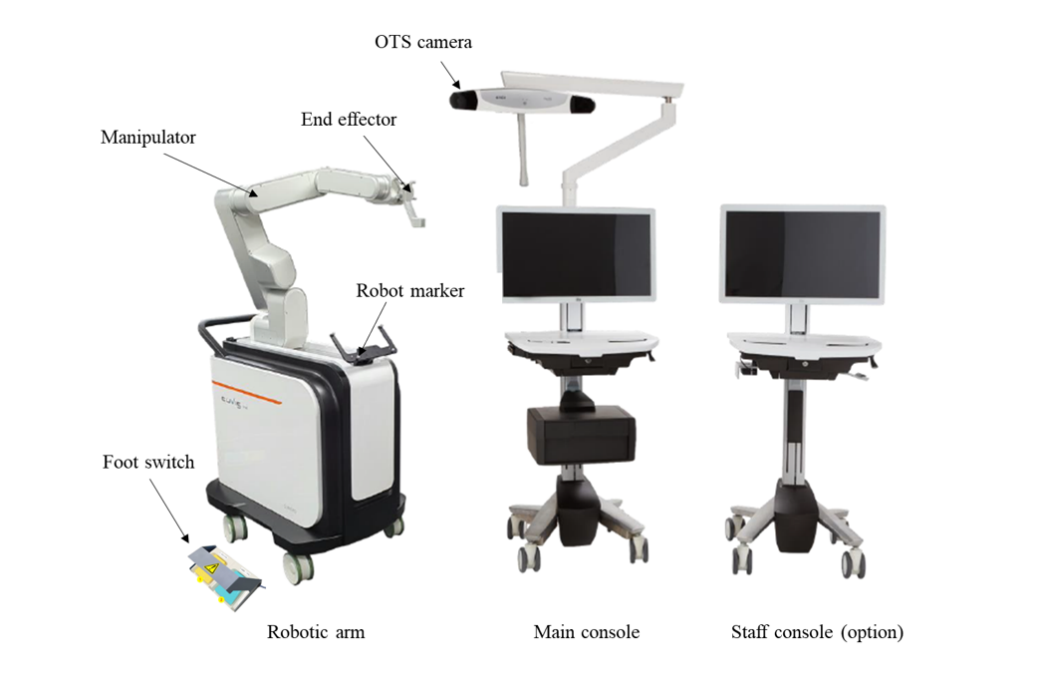
The CUVIS spine robot system: robotic arm (left), main console with navigation optical infrared tracking camera (middle), and the control workstation (right). OTS: optical tracking system.

Surgical navigation systems are used to plan the procedure and guide the surgeon in inserting the screws [1]. Robot spine surgery is popularly used to increase the accuracy of inserting screws in the spine, and the first robot used in spine surgery was the Spine Assist (Mazor Surgical Technologies), which received Food and Drug Administration clearance in 2004 [4]. The third-generation Mazor X system was cleared by the Food and Drug Administration in 2016 and, compared to previous generations, has a robotic arm that is attached to the patient’s body and can be viewed through a camera to ensure that the screws are inserted and the robotic arm is moving well [5]. The Mazor X Stealth Edition technology, which adds real-time image guidance and navigation during surgery, was cleared in 2019 and combines the best of both worlds: traditional spinal robotic surgery guidance and real-time software confirmation [5,6]. Figure 1 shows the evaluation device, which consists of a robotic arm, main console, and optional staff console, and is manufactured in South Korea. Like the Mazor X Stealth Edition technology, this product is capable of real-time image guidance and navigation during surgery.

### Usability

According to IEC (International Electrotechnical Commission) 62366-1 [7], usability has the following meaning: a “characteristic of the user interface that facilitates use and thereby establishes effectiveness, efficiency and user satisfaction in the intended use environment.” ISO (International Organization for Standardization) 9241-210 [8] defines usability as the “extent to which a system, product or service can be used by specified users to achieve specified goals with effectiveness, efficiency and satisfaction in a specified context of use.” These documents demonstrate that good usability in medical device design is essential to preventing error-related risks.

Evaluating usability focuses on determining whether the test device is easy for users to use. To evaluate the user experience of the device, we used usability tests and surveys for effectiveness, efficiency, satisfaction, and workload. The usability test was mainly used to identify use errors and efficiency, while the After-Scenario Questionnaire (ASQ) was conducted to evaluate the satisfaction of each task. NASA Task Load Index (NASA TLX) was used to measure the workload of the device, and System Usability Scale (SUS) was used to check the overall system usability of the device. Both ASQ and SUS identified the satisfaction and efficiency of the device, but in this study, ASQ identified the efficiency and satisfaction of each scenario, while SUS evaluated the satisfaction and efficiency of the device in the overall workflow.

## Methods

### Recruitment

We recruited 30 medical staff from Severance Hospital in South Korea. There were 15 doctors and 15 nurses. The intended users of the device are doctors and operating room nurses. Due to the different tasks that doctors and nurses have to perform when using the device overall, both groups were selected to participate in the usability test. We recruited through recommendations from colleagues and notice on the bulletin board at the Future Medicine Research Center at Gangnam Severance Hospital. Doctors and nurses who have experience using a robotic surgical device or navigation system were selected. For doctors, we selected those with the necessary knowledge of spine surgery, and for nurses, we selected those with experience in the operating room. However, those who had worked in the operating room for less than 1 year were excluded. After confirming these inclusion and exclusion criteria, the screening was conducted.

### Testing Procedure

One participant per session participated in the usability test, with the participant completing the assessment in a sequence guided by a facilitator, and an observer in the observation room videotaping the assessment and completing an observation sheet. A total of 2 moderators and 2 observers participated in the evaluation, with one of the moderators acting as a nurse if a doctor participated in the evaluation, and one of the moderators acting as a doctor if a nurse participated in the evaluation. In addition, observers were used to reduce bias by having 2 observers observe the usability test to ensure that one person’s opinion was not biased.

The facilitator introduced the participants to the usability test, obtained their informed consent, and trained them on the device for 20 minutes. Participants were allowed to interact with the device as much time as they needed. Afterward, 15 minutes were allowed between the training and evaluation to ensure that the training did not directly influence the evaluation [9]. Doctors and nurses were given different tasks because of the different job duties they do when operating with assessment devices. The tasks given to nurses focus on doctors’ instructions from preoperative preparation to surgery, while doctors focus on the surgery itself rather than preparing devices.

Participants then completed the evaluation for 40 minutes, with 8 scenarios (24 tasks) for doctors and 12 scenarios (41 tasks) for nurses. Doctors were asked to complete the following scenarios: preparation for use, preplanning of surgery, fixation of patient marker, scan, registration, verification, revisions of surgical planning, and navigation; nurses were asked to complete the following scenarios: preparation for use, system operation, initializing manipulator, drape, preparation of surgery, scan, registration, verification, planning of surgery, navigation, use of the emergency stop switch, and cleaning up after surgery. Tasks for each scenario are shown in Tables 1 and 2.

**Table 1:** Use scenarios of doctors.

Use scenario/task number		Task description
**Preparation for use**		
	Task 1	Check the contents related to the emergency button, foot switch, and foot jamming in the user manual.
	Task 2	Check the hand jamming label on the robotic arm manipulator.
**Preplanning of surgery**		
	Task 3	Select the spine level as follows: L4-L, L4-R L5-L, L5-R
	Task 4	After loading the first CT^a^ data, check the CT data.
	Task 5	Create an implant screw insertion path for target L4-L and L4-R and change the insertion path by moving the screw in the MPR^b^ view.
	Task 6	Create an implant screw insertion path for target L5-L and L5-R and change the insertion path using the arrow.
	Task 7	Check for collision between each screw.
**Fixation of patient marker**		
	Task 8	Attach the patient marker to the patient.
**Scan**		
	Task 9	Attach the registration tool adapter to the end effector.
	Task 10	After activating the hand guide function by pressing the AP^c^ button, change the position of the end effector according to the guidance on the pop-up window.
	Task 11	For C-Arm scan, attach the source calibrator to the end effector in the direction of AP and move the end effector to enable tracking by OTS^d^.
	Task 12	Check if the ROI^e^ includes the calibration marker, and the position and direction of the letters “R,” “G,” and “J” match the image, and then check pass or fail of registration.
**Registration**		
	Task 13	Perform segmentation to distinguish the surgical target in the image. L4-L, L4-R L5-L, L5-R
	Task 14	Perform labeling to assign target level information of ROI of 3D image and 2D image segmented for each spine level.
	Task 15	Adjust the ROI box so that the ROI of target L4 covers all the L4 vertebra area.
	Task 16	Perform 2D and 3D image registration for each spine level.
**Verification**		
	Task 17	After adjusting the CT image to overlay appropriately for target L4, check the registration result using the preview button and select whether to approve it.
	Task 18	After selecting whether to approve for target L5, perform image registration again so that the ROI includes all the vertebra area.
**Revision of surgical planning**		
	Task 19	Check the plan on 2D and 3D images, respectively.
	Task 20	As a result of planning for the entire target, check whether the robot can move in an area.
**Navigation**		
	Task 21	Insert the screw of target L4-L.
	Task 22	Through the [PRE-OP] screen, indicate the values for the insertion depth of the L4-L tapper, the amount of force applied to the end effector, and the patient’s movement.
	Task 23	Through the [INTRA-OP] screen, indicate the values for the insertion depth of the L4-L screw, the amount of force applied to the end effector, and the patient’s movement.
	Task 24	Move the end effector to the ready position for screw insertion to the target L4-R.

^a^CT: computed tomography.

^b^MPR: multiplanar reconstruction.

^c^AP: anterior-posterior.

^d^OTS: optical tracking system

^e^ROI: region of interest.

**Table 2:** Use scenarios of nurses.

Use scenario/task number		Task description
**Preparation for use**		
	Task 1	Check the contents related to the emergency button, foot switch, and foot jamming in the user manual.
	Task 2	Check the hand jamming label on the robotic arm manipulator.
	Task 3	Check if there are any abnormalities in the exterior of the robot marker frame and robotic arm.
	Task 4	Place the main console and staff console in a convenient location during surgery.
	Task 5	After checking the device and accessories in the operating room, assemble the marker ball.
	Task 6	Assemble the surgical tools such as the tapper’s driver and marker.
	Task 7	Assemble the surgical tools such as screwdriver and marker.
	Task 8	Assemble the clamp to be used to fix the patient marker.
**System operation**		
	Task 9	Connect the power and cables of the robotic arm, main console, and staff console.
	Task 10	After connecting the foot switch of the robotic arm, turn on the power of the robotic arm.
**Initializing manipulator**		
	Task 11	After logging in, select the surgical method and imaging device.
	Task 12	Check the connection status of the foot switch.
	Task 13	After selecting the robot position as “right,” initialize the manipulator (required to check movement notification sound and operation LED^a^).
	Task 14	Verify that the line laser on the robot marker intersects the area within range (required to check movement notification sound and operation LED).
**Drape**		
	Task 15	Follow the on-screen instructions to drape the patient to prevent infection (proceed in order of manipulator drape, base drape, and robot marker drape).
	Task 16	After installing the end effector of the robotic arm, assemble the marker ball where the robot marker drape is installed.
	Task 17	Move the manipulator to the ready position.
**Preparation for surgery**		
	Task 18	Move the robotic arm for patient surgery.
	Task 19	Check the surgical tools through OTS^b^, and if all surgical tools are not checked by the OTS camera, check if they are within the operating area.
	Task 20	The robot marker is not being recognized by the OTS camera due to damage to the marker ball. Replace with a new marker ball.
	Task 21	Please load the surgical data.
**Scan**		
	Task 22	Check if the ROI^c^ includes the calibration marker, and the position and direction of the letters “R,” “G,” and “J” match the image, and then check pass or fail.
**Registration**		
	Task 23	Perform segmentation to distinguish the surgical target in the image. L4-L, L4-R L5-L, L5-R
	Task 24	Perform labeling to assign target level information of ROI of the 3D image and 2D image segmented for each spine level.
	Task 25	Adjust the box so that the ROI of target L4 covers all of the vertebra area.
	Task 26	Perform 2D and 3D image registration for each spine level.
**Verification**		
	Task 27	Use the slide control at the bottom of the image to check whether the 2D and 3D images match to check the registration result.
	Task 28	For target L4, move the CT^d^ (DRR^e^) image by using the triangular button to adjust the 2 body images to be similar.
	Task 29	For target L4 whose registration result has been adjusted, use the preview button to check the registration result and select whether or not to approve it.
	Task 30	After selecting whether or not to approve for target L5, perform image registration again so that the ROI includes all of the vertebra area (target L5: registration failed).
	Task 31	After displaying the planned data on the screen through the preview button for each target for which the registration result has been adjusted, check if the registration is completed normally.
	Task 32	Depending on the registration result of target L5, select whether or not to approve (target L5: registration completed normally).
**Planning of surgery**		
	Task 33	Check whether the robot can move to the planned position.
**Navigation**		
	Task 34	On the screen, move the end effector of the robotic arm to the planned guide position relative to target L4-L.
	Task 35	Move the end effector to the original position for the guide.
	Task 36	On the screen, move the end effector of the robotic arm to the planned guide position relative to target L4-R.
**Use of the emergency stop switch**		
	Task 37	(At the moment, the manipulator is positioned too close to the patient.) Press the emergency button.
	Task 38	Release the emergency button.
**Cleaning up after surgery**		
	Task 39	Shut down the main console.
	Task 40	Shut down the robotic arm.
	Task 41	Disconnect the cable.

^a^LED: light emitting diode.

^b^OTS: optical tracking system.

^c^ROI: region of interest.

^d^CT: computed tomography.

^e^DRR: digitally reconstructed radiograph.

**Figure 2:**
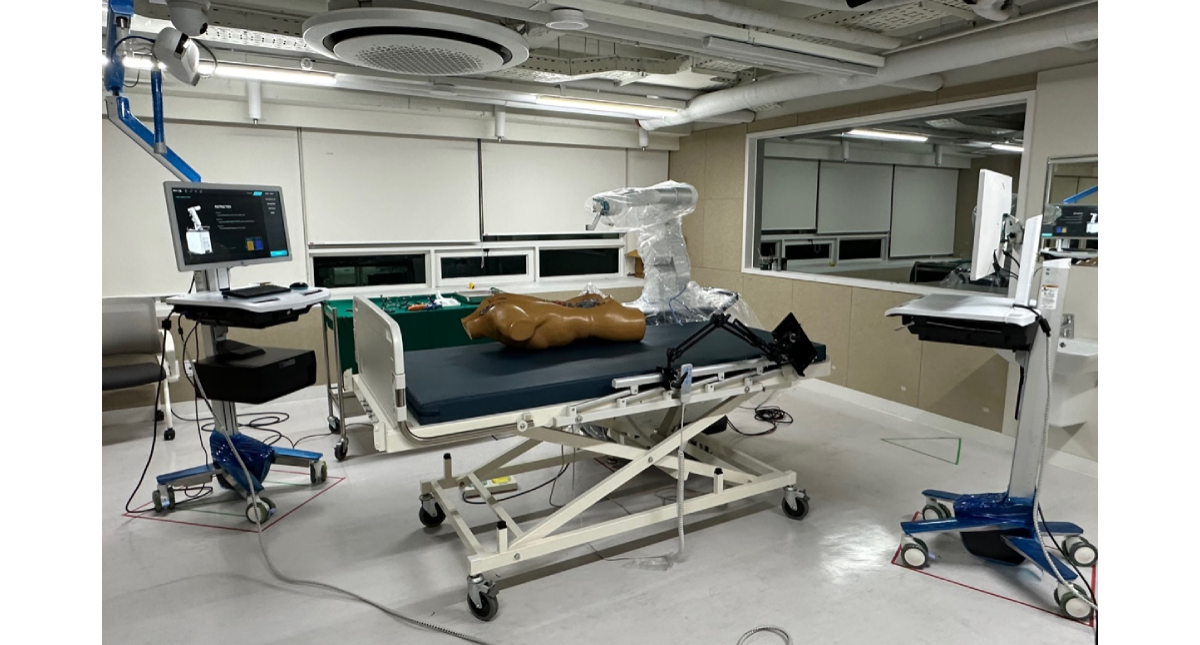
Test environment: the simulated environment is organized to resemble the operating room in which the evaluator is used.

The test environment as shown in Figure 2 is organized to resemble the operating room. Participants used the device following prompts presented on a stand monitor. The test environment was organized similar to an operating room, considering the use environment of the robot spine surgery. An operating room bed, an upper torso dummy, and a patient monitoring device were prepared similar to the actual operating room environment. The temperature and humidity of the evaluation room were measured and recorded right before the evaluation. Similar to a real operating room, the temperature was kept between 20 °C and 24 °C, and the humidity was between 30% and 60%.

**Figure 3:**
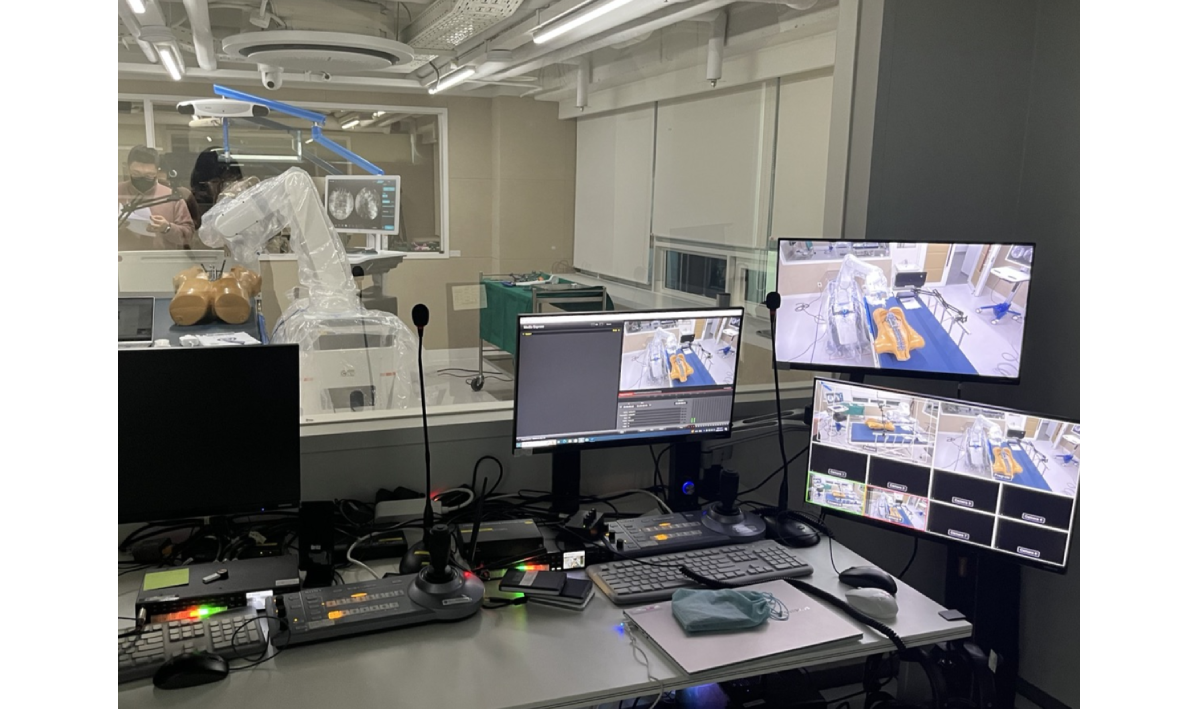
Test observation environment: we set up monitoring equipment to observe and record the entire evaluation process in real time.

The evaluation facilitator guided the participant if they requested assistance with a use scenario, and an observer recorded all participant interactions from outside the test room with a 1-way mirror. The test observation environment setting is shown in Figure 3. The observer used a program from Media Express to record the progress of the usability evaluation. At the end of the evaluation, 3 types of questionnaires were administered.

### Statistical Analysis

#### ASQ Measure

After each scenario, the participants completed the ASQ created by Lewis [10] and developed from the ISO 9241-11 standard questionnaire [11]. This is one of the most popular surveys for assessing usability because it is the simplest and its 3 items are easy for participants to understand [11]. As shown in Textbox 1, the ASQ consists of 3 questions, each corresponding to the user’s satisfaction with the ease, efficiency according to the time taken to complete the scenario, and validity of the information provided. Participants responded to each question on a 7-point Likert scale [10,12]. Participants rated their satisfaction about the device’s usability based on each task scenario [13]. A score of “1” means strongly disagree, and a score of “7” means strongly agree [14]. We found the mean and SD for the 3 questions participants asked ASQ.

**After-Scenario Questionnaire (ASQ).**

ASQ1: Overall, I am satisfied with the ease of completing the tasks in this scenario.

ASQ2: Overall, I am satisfied with the amount of time it took to complete the tasks in this scenario.

ASQ3: Overall, I am satisfied with the support information (digital help, messages, and documentation) when completing the tasks.

#### NASA TLX Measure

**Figure 4:**
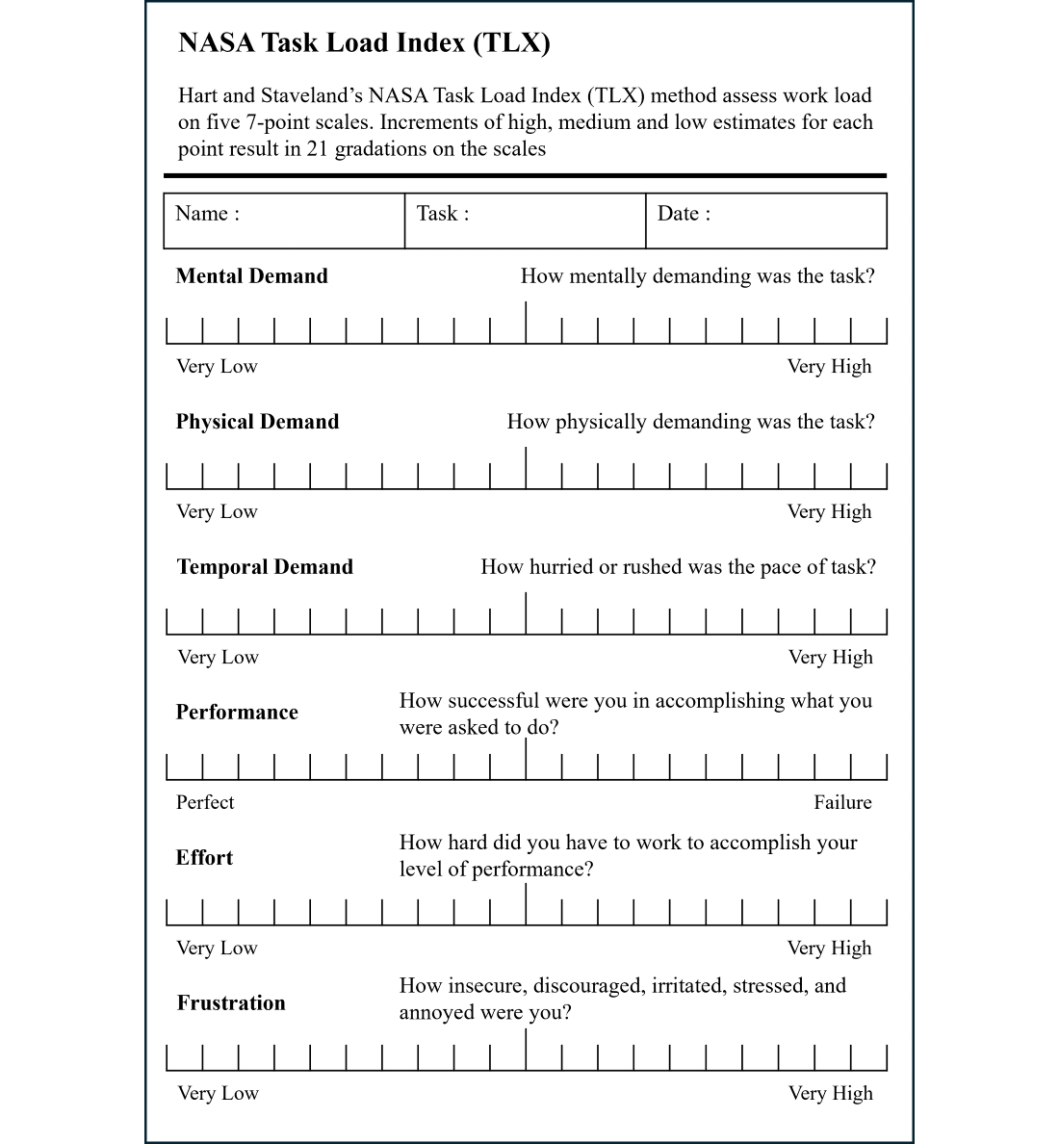
NASA Task Load Index.

NASA TLX measures cognitive workload, and like usability, workload is a complex construct that determines the amount of physical and mental effort required to use an interface [15,16]. The workload is assessed by the US NASA TLX [15,17]. The most effective way to assess a worker’s perceived job difficulty is to ask questions directly to workers who have experienced the job. As shown in Figure 4, the Task Load Index (TLX) uses 6 dimensions to measure workload. The 6 metrics are mental demand, temporal demand, physical demand, performance, effort, and frustration [16,18]. The NASA TLX scores are evaluated by dividing the score into 21 steps, subtracting 1 from the score, and multiplying it by 5 to express it on a scale of 0 to 100 [19,20]. On a scale of 100, when the score is lower, the workload is lower. Less work means a less complex and easier-to-use user interface. On a 100-point scale, the workload can be described as low (0-9), medium (10-29), rather high (30-49), high (50-79), and very high (80-100) [21]. In the NASA TLX, performance assesses satisfaction with task completion, with the lowest number representing perfect and the highest number representing failure [22]. The point system for mental, physical, temporal, effort, and frustration part ranges from very low to very high [15,16,19,22,23].

#### SUS Measure

As shown in Textbox 2, the SUS consists of 10 items that assess the participant’s level of agreement with the overall usability of the system, with odd-numbered items being positive and even-numbered items being negative [24]. SUS is the most commonly used usability assessment questionnaire [24]. Participants responded to each item on a 5-point Likert scale [13]. The scale ranges from 1=strongly disagree to 5=strongly agree [25,26]. To calculate the SUS score from the points acquired from the 5-point Likert scale, the following subtractions were used. For odd-numbered items, subtract 1 from the user response, and for even-numbered items, subtract the user responses from 5. With this calculation, the value range changes from 0 to 4. The most positive response is 4. The scores from each converted response were multiplied by 2.5 to a total possible point of 100. The SUS is percentage-based and divided into 5 levels: A (>80.3), B (68-80.3), C (68), D (51-68), and F (<51) [24]. A score of 85 is considered very good usability, and a score of 68-84 is considered good usability [25,26].

**System Usability Scale (SUS) items.**

SUS1: I think that I would like to use this system frequently.

SUS2: I found the system unnecessarily complex.

SUS3: I thought the system was easy to use.

SUS4: I think that I would need the support of a technical person to be able to use this system.

SUS5: I found the various functions in this system were well integrated.

SUS6: I thought there was too much inconsistency in this system.

SUS7: I would imagine that most people would learn to use this system very quickly.

SUS8: I found the system very cumbersome to use.

SUS9: I felt very confident using the system.

SUS10: I needed to learn a lot of things before I could get going with this system.

#### Data Analysis

ASQ, NASA TLX, and SUS results were computed using SPSS (version 22; IBM Corp) [27]. Descriptive statistics were performed on for doctor and nurse characteristics. Doctors and nurses were compared on age, gender, work experience, and use experience with similar devices. For the questionnaire items, values were compared between groups using 2-tailed *t* tests for normality and Mann-Whitney *U* tests for nonparametric tests. Figures are presented as the mean and SD, and *P*<.05 was considered significant.

### Ethical Considerations

This study was approved by the institutional review board of Yonsei University Health System, Gangnam Severance Hospital (3-2022-0493). All the participants who passed the screening signed an informed consent form. Furthermore, all information collected about the participants was anonymized. This study complied with the Code of Ethics. Participants received monetary compensation for participating in the evaluation.

## Results

### User Statistics

In total, 15 doctors and 15 nurses, each representing the intended users of the surgical assistant, participated in the evaluation. Participants were recruited from doctors and nurses at Severance Hospital. Table 3 shows the sociodemographic characteristics of participants. Both doctors and nurses were between the ages of 30 and 39 years. For both doctors and nurses, those with different experience levels were recruited, and opinions were collected from all the participants. In particular, those with more experience with surgical devices were able to gather relevant opinions because they were more familiar with the device or the existing surgical methods, while those with less experience focused on whether the device was easy to use without much experience. Doctors’ professional experience ranged from 2 to 20 years, with an average of 7.53 (SD 5.45) years of professional experience. The nurses’ professional experience ranged from 5 to 26 years, with an average of 12.93 (SD 6.43) years. The surgical assistants had used Medtronic (Medtronic), Stryker (Stryker Corp), and Curexo (Curexo, Inc), with an average of 3 (SD 2.36) years of experience.

**Table 3:** Sociodemographic characteristics and experience of the test participants (N=30).

Variable			Group 1 (doctors), n (%)	Group 2 (nurses), n (%)
**Sociodemographic characteristics**				
	**Age (years)**			
		20-29	3 (20)	1 (7)
		30-39	10 (67)	8 (53)
		40-49	1 (7)	6 (40)
		50-59	1 (7)	0 (0)
	**Sex**			
		Male	14 (93)	6 (40)
		Female	1 (7)	9 (60)
	**Work experience**			
		More than 1 year, less than 5 years	7 (47)	0 (0)
		More than 5 years, less than 10 years	3 (20)	4 (27)
		More than 10 years, less than 15 years	3 (20)	6 (40)
		More than 15 years, less than 20 years	1 (7)	1 (7)
		More than 20 years	1 (7)	4 (27)
**Use experience with similar devices**				
	**Device name**			
		Medtronic	12 (80)	7 (47)
		Stryker	1 (7)	0 (0)
		Curexo	2 (13)	8 (53)
	**Use experience**			
		Less than 1 year	6 (40)	3 (20)
		More than 1 year, less than 3 years	5 (33)	8 (53)
		More than 3 years, less than 5 years	3 (20)	4 (27)
		More than 5 years, less than 10 years	0 (0)	0 (0)
		More than 10 years	1 (7)	0 (0)

### Task Completion

The 15 doctors performed 24 tasks within 8 large scenarios, while the nurses performed a total of 41 tasks within 12 scenarios. As shown in Table 4, for the doctors, all 8 scenarios had a success rate of 90% or higher, with the lowest success rate for the revising a surgical plan scenario. As shown in Table 5, for nurses, all 11 scenarios except the surgical plan had a success rate of 90% or higher, with the planning of surgery scenario having an 87% success rate.

**Table 4:** Task completion rate in doctors.

	Task pass rate (%)	Task failure rate (%)
Preparations for use	96.67	3.33
Preplanning of surgery	98.67	1.33
Fixation of patient marker	100	0
Scan	96.67	3.33
Registration	100	0
Verification	96.67	3.33
Revision of surgical planning	93.33	6.67
Navigation	98.33	1.67

**Table 5:** Task completion rate in nurses.

	Task pass rate (%)	Task failure rate (%)
Preparations for use	97.50	2.5
System operations	96.67	3.33
Initialization manipulator	100	0
Drape	93.33	6.67
Preparation for surgery	91.67	8.33
Scan	100	0
Registration	98.33	1.67
Verification	91.11	8.89
Revision of surgical planning	86.67	13.33
Navigation	100	0
Use of the emergency stop switch	100	0
Cleaning up after surgery	95.56	4.44

Among the scenarios in which the doctors did not successfully complete a task during the assessment, the critical tasks were as follows: in task 11, the participant failed to follow the process of attaching the source calibrator in the opposite direction to track the optical tracking system and did not recognize the correct attachment method. In addition, pressing the anterior-posterior scan button and moving the end effector closer to the dummy proceeded correctly, but before attaching the source calibrator, the optical tracking system process could not be performed because it did not proceed in the existing pop-up window and proceeded to the data acquisition step. In task 18, participants selected the target but were unable to click the “Re-matching” button. To proceed with rematching, a target needs to be selected and pressed, but the Re-matching button could not be clicked because the target was not selected. In task 21, the participant did not recognize whether the robot movement was completed by continuously pressing the foot switch without releasing it. In this case, the participant said that he was unable to perform the task because there was no indication on the screen that the robot’s movement was complete, and there was no visual or audible user interface.

The nurse was unable to complete task 28 due to difficulty using the image adjustment feature. Participants were asked to move the computed tomography image and adjust the body image to be similar but could not comprehend how to use the “Adjustment” function or the “Re-matching” function (the user did not recognize the intended function itself). Even when the “Adjustment” function was used, it was observed that the user could not use the “Adjustment” function in the way intended by trying to adjust the overlayed screen itself rather than adjusting the screen by pressing the button. If an accurate match is not made, the manipulator may move to a different location than the user’s target location, causing potential harm. In addition, nurses had difficulty using the reassembly feature of task 30. The “Re-matching” function could not be used because the target was released while pressing the “Disapproved” button in the rematching task, or the “Adjustment” function was used rather than using the “Re-matching” function. Participants failed to perform the task because they did not recognize that the “Re-matching” function could only be used by resetting the target that was released when pressing the “Disapproved” button, or that “Re-matching” meant rematching. This caused potential harm by moving the manipulator to a location different from the user’s target location. Nurses were unsuccessful in tasks such as registration, verification, and navigation because these tasks are usually performed through doctors’ orders. During the scenarios, there were no given orders, forcing the nurses to make their own decisions, which they are not accustomed to.

Overall, 4 doctors said that when creating a screw position in the planning stage, the position is created in a completely different part from the actual location; thus, it would be better if the position could be created closer to the target, and when moving the position, that it would be better to be able to check other position paths at the same time. In total, 7 doctors said it would be better if there was notification or guidance for the arrival of the robot arm at the target so that moving to the guided position can be recognized. In addition, 5 doctors and 8 nurses found that in the overall process of selecting and adjusting the region of interest (ROI) box to the target area, it was inconvenient to select and release the box, and that it was difficult to adjust because of its excessive rotation.

### Usability (ASQ)

After the usability evaluation, doctors and nurses were surveyed using the ASQ for each scenario. For both doctors and nurses, the ASQ for registration was divided into 2 parts: first, segmentation and labeling, and second, ROI setting and image matching. As shown in Tables 6 and 7, among the registration items, both doctors and nurses had the lowest scores for the ROI setting and image matching, followed by ease of completing the task (group 1: mean 4.73, SD 1.57 and group 2: mean 4.47, SD 1.89), amount of time it took (group 1: mean 4.47, SD 1.63 and group 2: mean 4.40, SD 2.09), and support information satisfaction (group 1: mean 5.13, SD 1.50 and group 2: mean 5.13, SD 1.89). The doctors’ opinions were mainly that it was inconvenient to have to click on the line precisely; thus, the ease of adjustment should be improved. Nurses reported that they were less sensitive to the 360-degree rotation button at the top of the ROI box and had difficulty clarifying the image while adjusting the ROI box.

**Table 6:** After-Scenario Questionnaire result in group 1 (doctors).

	Ease of completing the task, mean (SD)	Amount of time it took, mean (SD)	Support information satisfaction, mean (SD)
User manual	5.56 (1.12)	5.54 (1.28)	5.63 (1.19)
Preplanning of surgery	5.80 (0.98)	5.53 (1.26)	5.93 (0.77)
Fixation of patient marker	6.00 (0.97)	6.40 (0.61)	—^a^
Scan	6.00 (0.63)	5.60 (0.95)	5.93 (0.77)
Registration 1 (segmentation and labeling)	5.73 (1.18)	5.07 (1.53)	6.00 (0.82)
Registration 2 (ROI^b^ setting and image matching)	4.73 (1.57)	4.47 (1.63)	5.13 (1.50)
Verification	5.60 (1.14)	5.40 (1.31)	5.73 (1.00)
Revision of surgical planning	5.80 (0.83)	5.93 (0.68)	5.93 (1.29)
Navigation	5.67 (1.07)	5.93 (0.77)	5.33 (1.45)

^a^Not available; fixation of patient markers was not surveyed because they do not have any on-screen information.

^b^ROI: region of interest.

**Table 7:** After-Scenario Questionnaire result in group 2 (nurses).

	Ease of completing the task, mean (SD)	Amount of time it took, mean (SD)	Support information satisfaction, mean (SD)
User manual	6.00 (1.23)	5.80 (1.63)	5.97 (1.23)
Preparations for use	6.47 (0.62)	6.07 (1.12)	6.40 (1.02)
System operations	6.27 (0.85)	6.20 (0.98)	6.2 (1.11)
Initialization manipulator	6.13 (1.15)	5.87 (1.45)	5.87 (1.45)
Drape	5.27 (1.84)	5.67 (1.62)	5.80 (1.51)
Preparation for surgery	6.13 (0.88)	6.07 (0.93)	6.27 (0.93)
Scan	6.07 (0.85)	5.87 (0.88)	6.07 (1.06)
Registration 1 (segmentation and labeling)	5.53 (1.50)	4.93 (1.81)	5.67 (1.85)
Registration 2 (ROI^a^ setting and image matching)	4.47 (1.89)	4.40 (2.09)	5.13 (1.89)
Verification	4.93 (1.77)	5.20 (1.38)	5.40 (1.74)
Navigation	6.60 (0.61)	6.60 (0.61)	6.60 (0.61)
Use of the emergency stop switch	6.73 (0.44)	6.60 (0.61)	6.73 (0.44)
Cleaning up after surgery	6.47 (0.88)	6.40 (1.02)	6.40 (1.02)

^a^ROI: region of interest.

Tables S1 and S2 in Multimedia Appendix 1 show categorization by use experience with similar devices. The ASQ results did not show significant differences in satisfaction based on use experience and years of experience with robotic surgical systems. For doctors, those with more than 3 years of experience using robotic surgical systems found it easier and faster to perform tasks. For nurses, participants with more experience using similar devices scored higher than those with less than 3 years of experience on the need to prepare before surgery.

### Workload (NASA TLX)

Table 8 shows the results of the workload of the assistive robotic surgery devices by occupational group for mental demand, physical demand, temporal demand, performance, effort, frustration, and overall TLX. The Mann-Whitney *U* test comparing the TLX scores of the doctors and nurses, including mental demand (*P*=.81), physical demand (*P*=.90), temporal demand (*P*=.87), performance (*P*=.81), and frustration (*P*=.81) and the independent 2-sample *t* test comparing the TLX scores of effort (*P*=.64) and overall TLX (*P*=.77) showed no significant differences in the scores. Doctors’ workload levels were generally in the medium (10-29), and nurses were also in the medium (10-29) except for effort.

**Table 8:** Result of NASA Task Load Index.

	Group 1^a^ (n=15), mean (SD)	Group 2^b^ (n=15), mean (SD)	*t* test^c^ (*df*)	*U* test^d^	*P* value^e^
Mental demand	30.67 (25.97)	29.33 (29.39)	N/A^f^	106.5	.81
Physical demand	13.00 (15.09)	11.33 (12.17)	N/A	109.5	.90
Temporal demand	15.00 (17.63)	22.33 (30.58)	N/A	108.5	.87
Performance	25.00 (28.09)	28.33 (26.16)	N/A	106	.81
Effort	29.67 (24.60)	34.33 (28.78)	–0.477 (28)	N/A	.64
Frustration	19.00 (22.22)	18.33 (26.70)	N/A	106	.81
Overall Task Load Index	22.06 (18.58)	24.00 (16.96)	–0.299 (28)	N/A	.77

^a^Group 1: doctors.

^b^Group 2: nurses.

^c^Because the data were normally distributed, a independent 2-sample *t* test was used.

^d^Because the data were not normally distributed, the Mann-Whitney *U* test was performed.

^e^*P* values were determined with the independent 2-sample *t* test and Mann-Whitney *U* test for continuous variables.

^f^N/A: not applicable.

**Figure 5:**
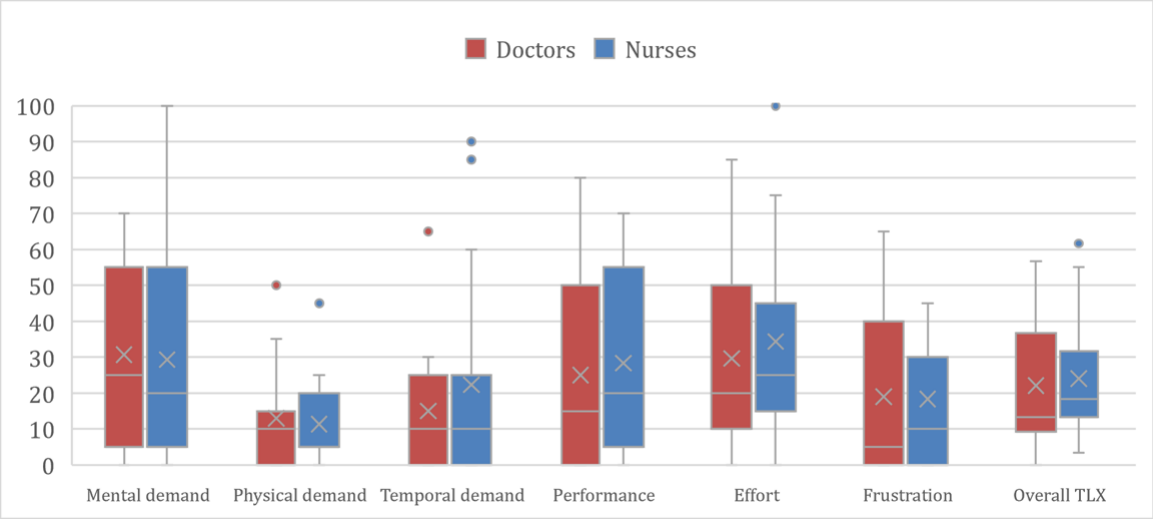
Workload results by group: distribution of the NASA TLX scores for doctors and nurses. TLX: Task Load Index.

**Figure 6:**
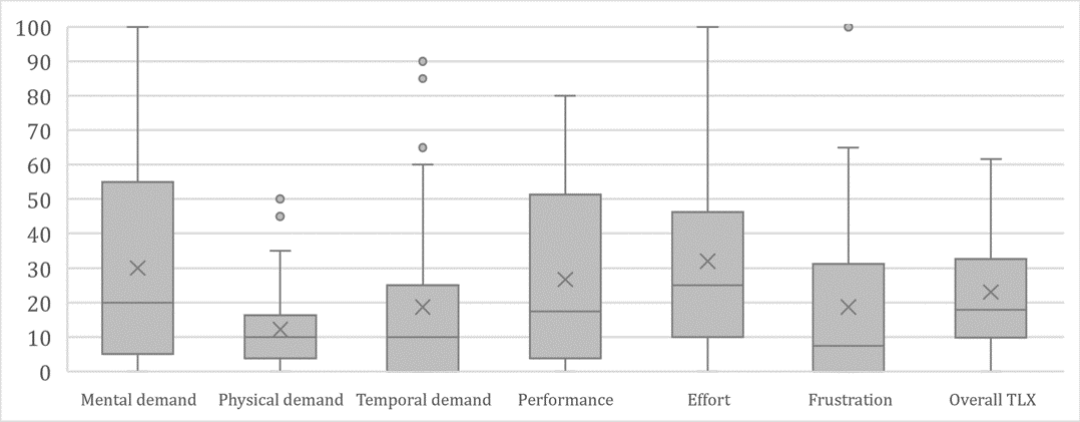
Workload results: distribution of the overall NASA TLX scores. TLX: Task Load Index.

Figure 5 shows a boxplot of the NASA TLX results for doctors and nurses. In addition, Figure 6 shows the NASA TLX results for all evaluation participants (doctors and nurses). The box plots show the maximum (45-100), median (5-25) minimum (0), first quartile (0-17.5), and third quartile (12.5-52.5), with the center box showing the median of 50% of the cases. When comparing the workload of the doctors and nurses, there was no significant difference as shown in Table 8, and we can see that 3 categories, physical demand, temporal demand, and frustration, have lower workloads than the others.

In Tables 9 and 10, NASA TLX scores were compared based on use experience with similar devices. Participants who had been using robotic surgical systems for more than 3 years, both doctors and nurses, reported that the evaluation device required a lot of effort to use. Participants had difficulty using the evaluation device because it was more complex than similar robotic surgical devices. However, when it comes to temporal demand, those who have been using robotic surgical systems for more than 3 years reported that it is not time-consuming. The workflow of the evaluation device is not much different from existing robotic surgical devices, and the graphical user interface is easy to use and can be performed quickly.

**Table 9:** Comparison of the NASA Task Load Index by use experience of doctors.

	All, mean (SD)	Less than 3 years, mean (SD)	More than 3 years, mean (SD)
Mental demand	30.67 (25.97)	27.00 (28.21)	38.00 (21.68)
Physical demand	13.00 (15.09)	14.00 (16.12)	11.00 (14.32)
Temporal demand	15.00 (17.63)	18.50 (19.87)	8.00 (10.37)
Performance	25.00 (28.09)	22.50 (28.80)	30.00 (29.15)
Effort	29.67 (24.60)	21.50 (19.59)	46.00 (27.48)
Frustration	19.00 (22.22)	15.50 (20.20)	26.00 (26.79)

**Table 10:** Comparison of the NASA Task Load Index by use experience of nurses.

	All, mean (SD)	Less than 3 years, mean (SD)	More than 3 years, mean (SD)
Mental demand	29.33 (29.39)	25.91 (22.56)	38.75 (46.61)
Physical demand	11.33 (12.17)	12.27 (12.72)	8.75 (11.81)
Temporal demand	22.33 (30.58)	28.64 (33.70)	5.00 (5.77)
Performance	28.33 (26.16)	22.27 (24.73)	45.00 (25.50)
Effort	34.33 (28.78)	30.45 (25.73)	45.00 (38.08)
Frustration	18.33 (26.70)	15.45 (15.40)	26.25 (49.22)

### Usability (SUS)

Both doctors and nurses who participated in the usability test completed the SUS questionnaire. Table 11 shows that the mean score of SUS was 64.17 (SD 16.52) for doctors and 61.67 (SD 19.18) for nurses. The nonparametric Mann-Whitney *U* test was used to analyze *U* values and *P* values presented in Table 11. When comparing the SUS scores of the doctors and nurses (*P*=.84, greater than .05), we can see that there was no significant difference between the 2 values. Figure 5 is a boxplot comparing the SUS scores of the doctors and nurses, showing a baseline of 68, which is the average SUS score. For the nurses and doctors, this corresponds to a grade of D on the SUS scale.

**Table 11:** Result of the System Usability Scale (SUS).

	Group 1^a^, mean (SD)	Group 2^b^, mean (SD)	*U* test^c^	*P* value^d^
SUS1: I think that I would like to use this system frequently.	2.33 (1.14)	2.60 (1.14)	96.5	.51
SUS2: I found the system unnecessarily complex.	2.4 (1.02)	2.67 (1.25)	92.5	.41
SUS3: I thought the system was easy to use.	2.93 (0.77)	2.93 (1.00)	104	.74
SUS4: I think that I would need the support of a technical person to be able to use this system.	2.00 (1.32)	1.00 (0.97)	65	.05
SUS5: I found the various functions in this system were well integrated.	3.00 (0.82)	2.93 (1.06)	109.5	.90
SUS6: I thought there was too much inconsistency in this system.	2.67 (0.94)	2.47 (1.31)	111	.97
SUS7: I would imagine that most people would learn to use this system very quickly.	2.87 (0.88)	3.20 (1.11)	82	.22
SUS8: I found the system very cumbersome to use.	2.53 (1.09)	2.73 (1.06)	100.5	.62
SUS9: I felt very confident using the system.	2.87 (0.81)	2.73 (0.88)	65	.05
SUS10: I needed to learn a lot of things before I could get going with this system.	2.07 (1.18)	2.00 (1.10)	106.5	.81
Overall, SUS score on 0 to 100 normalized scale	64.17 (16.52)	61.67 (19.18)	107.5	.84

^a^Group 1: doctors.

^b^Group 2: nurses.

^c^Because data were not normally distributed, the Mann-Whitney *U* test was performed.

^d^*P* values were determined with an independent 2-sample *t* test and the Mann-Whitney *U* test for continuous variables.

When we compared participants’ use experience with the device between those who had used it for more than 3 years and those who had used it for less than 3 years, we found that for doctors, those who had used it for more than 3 years had lower SUS scores than those who had used it for less than 3 years. For doctors, the average SUS score for participants with 3 or more years of experience is 55.5 (SD 19.13), while the average SUS score for those with less than 3 years is 68.5 (SD 13.05). For nurses, similar to doctors, we found that participants who had used a similar device for more than 3 years had lower scores than those who had used it for less than 3 years, at 53.75 versus 64.55 (Table 12).

**Table 12:** System Usability Scale (SUS) comparison by use experience of a similar device.

	Group 1^a^				Group 2^b^		
	All, mean (SD)	Less than 3 years, mean (SD)	More than 3 years, mean (SD)	All, mean (SD)		Less than 3 years, mean (SD)	More than 3 years, mean (SD)
SUS	64.17 (16.52)	68.5 (13.05)	55.5 (19.13)	61.67 (19.18)		64.55 (15.14)	53.75 (25.77)

^a^Group 1: doctors.

^b^Group 2: nurses.

## Discussion

### Principal Findings

This study is a summative evaluation to examine the usability of the frameless stereotaxic navigation system (model CS200) that is used as an auxiliary tool for guiding the surgical tool to the target position and posture planned by the user in the incision or percutaneous spinal surgery. Regarding the use scenario, (1) task success, (2) use error, (3) satisfaction (ASQ), (4) workload (NASA TLX), and (5) SUS related to the usability of the test device were evaluated and analyzed. The usability test was conducted by professional medical staff who have completed specialized medical education and obtained professional medical qualifications. The participants in the usability test were doctors and nurses who have experience in using spinal surgery robots or navigation systems in operating rooms.

For doctors, all 8 scenarios had a success rate of at least 93% or higher, and for nurses, all 12 scenarios had a success rate of at least 87% or higher. Doctors had the lowest success rate of 93% on the “revision of surgical planning” scenario. In the ASQ results, the average score for “ease of completing the task” was 5.66 (SD 0.36), the average score for “amount of time it took” was 5.54 (SD 0.52), and the average score for “support information satisfaction” was 5.70 (SD 0.30). When performing the usability evaluation, the “revision of surgical planning” scenario had the lowest success rate; however, the 3 ASQ scores were higher than the average: 5.80 (SD 0.83), 5.93 (SD 0.68), and 5.93 (SD 1.29).

The “image matching” scenario had the lowest score for each item in the satisfaction score, even though it had a 100% success rate. We found that a high success rate on the evaluation task does not necessarily indicate high usability satisfaction. Despite the high task success rate in the usability test, the low satisfaction rate in the questionnaire that evaluated the usability aspects of the device indicates a lack of satisfaction with the device.

Although there were no difficulties in performance, the task of adjusting the ROI within the “registration” scenario was criticized for its difficulty in accurately adjusting the ROI and its lack of usability. Nurses, like doctors, had the lowest success rate of 87% in the “revision of surgical planning” scenario. However, the ASQ survey results for the “registration” scenario, which had the highest success rate of 98%, showed the lowest scores for “ease of completing the task” with a mean of 4.47 (SD 1.89), “amount of time it took” with a mean of 4.40 (SD 2.09), and “support information satisfaction” with a mean of 5.13 (SD 1.89). Similar to the doctors, when adjusting the ROI box, many of the nurses commented that the 360-degree rotation button at the top was not sensitive, and the video was difficult to see clearly. In addition, both doctors and nurses reported that when moving the robot arm using the footswitch during the “navigation” scenario, there was no visual or audible indication of how far the robot arm had moved and whether it had completed its movement, resulting in collisions between the robot arm and the patient stack. If used with real patients, this could lead to a significant risk of patient injury. We believe that the usability of these screens needs to be improved.

When comparing the workloads of the 2 groups who primarily use the assessment tool, we found that there was no difference, and that the workloads of mental demand and performance are high for both groups. Doctors and nurses commonly commented that the process was too complicated and laborious and that they had doubts about the accuracy of the ROI adjustment. In addition, since the robot assists in surgery, we thought it would be a quick process, but we found that the robot needed more time to move than expected, which affected the workload.

The SUS also showed no significant difference between doctors and nurses, with slightly lower-than-average satisfaction scores. The doctor gave the system a low score on the usability scale because it was too time-consuming to use in the actual operating room. Nurses gave low scores because of the time-consuming setup prior to actual surgery. Both doctors and nurses gave low scores, especially on the items that they felt they would need technician support to use the system and that they would need to learn about the system before its use, because many of them had never used an assessment device before and were not familiar with it. In addition, the lack of usability, with no explanation of what to do next on the device and no prompts to prevent errors, contributed to the low scores. At the hospital where participants work, engineers who have no difficulty using similar devices are present to aid doctors and nurses in using the device. Because of their reliance on engineers, many of the participants had difficulty in using the device alone and commented that they needed the engineers’ support. This suggests that the device needs to be highly usable with an easily understandable user interface and a screen design that is familiar to medical staff so that they can use the device without engineers’ assistance. Overall, when evaluating usability, there was no significant difference between participants who had used similar devices for more than 3 years and those who had used them for less than 3 years, except for ASQ and SUS, which evaluate satisfaction, and NASA TLX, which evaluates the difficulty of the operator’s job. It was found that there were differences in job duties when using the equipment that they were familiar with as well as differences in the time taken to perform the tasks. In other items, there seems to be no problem in using the device once they are familiar with it.

Table S3 in Multimedia Appendix 1 shows the improvements made to the device since the usability evaluation. The user interface was improved by quantifying and intuitively displaying data that used to be shown only as graphs. Confusing highlighted buttons that hindered the use of the device were rearranged to decrease the errors made by users.

To further increase the usability of the device, the footswitch needs to be improved to recognize how much movement is required to move the device by displaying the information on the interface. In particular, during the navigation phase, it would increase the usability if a notification or on-screen guidance appeared when the target was reached while moving to the location guided by the robotic arm. In addition, the drape is divided into 3 stages, while other similar devices only have 1 drape, increasing the risk of contamination.

Although the participants have experience using third-party equipment, they had difficulty using this evaluation device for the first time because they were not familiar with it; however, we do not think there will be any major problems once they are accustomed to the evaluation device. In addition, as a robot that guides the position of the screw in the patient’s body, it should have a more accurate and simpler workflow, making it more competitive with other products.

### Limitations

This study is limited by the fact that our evaluation took place at a single institution, Severance Hospital in South Korea. In South Korea, robotic surgery is not yet widely used, and many people have not used robotic surgical instruments before; thus, they are still unfamiliar with robotic surgical instruments. However, the strength of this study is that we conducted usability tests with doctors and nurses in the operating room, who are the closest users of the new system, the surgical assistant robot.

### Conclusions

In group 1, a success rate of 93% or higher was observed in all 24 tasks. In group 2, a success rate of 87% or higher was observed in 38 of 41 tasks. A success rate of 80% was observed in the task related to marker ball view confirmation (task 18), 80% in the task related to the use of the “Adjustment” function (task 28), and 75% in the task related to using “Re-matching” (task 30).

In addition, subjective data such as follow-up questions and surveys were more effective in identifying shortcomings and judging the usability, satisfaction, and effectiveness of the device than quantitative data such as the number of use errors (task completion rate) and satisfaction evaluation scores. In terms of error, participants provided a lot of feedback, including suggestions for mitigating potential risks. Although the task success rate was high, the workload and SUS scores were lower than the baseline, suggesting that improving the device user interface would increase the usability of the system. We recommend that the results of this test can be used in other usability engineering processes to improve the overall usability, satisfaction, completeness, and efficiency.

## Appendix

Multimedia Appendix 1 After-Scenario Questionnaire detailed results for doctors and nurses.

## Abbreviations

ASQ: After-Scenario Questionnaire
IEC: International Electrotechnical Commission
ISO: International Organization for Standardization
NASA TLX: NASA Task Load Index
ROI: region of interest
SUS: System Usability Scale
TLX: Task Load Index
