# Supplementary material for: User Experience Evaluation of a Spinal Surgery Robot: Workload, Usability, and Satisfaction Study
Source: JMIR Hum Factors. 2024 Apr 1;11:e54425. doi: 10.2196/54425 (PMC11019418; doi:10.2196/54425)
Supplement: Multimedia Appendix 1 [file humanfactors_v11i1e54425_app1.docx]

# **Table S1.** ASQ result in doctors.

|  | Ease of completing the task Mean(SD) | | | Amount of time it took Mean(SD) | | | Support information satisfaction Mean(SD) | | |
| --- | --- | --- | --- | --- | --- | --- | --- | --- | --- |
|  | ALL | Less than 3 years | More than 3 years | ALL | Less than 3 years | More than 3 years | ALL | Less than 3 years | More than 3 years |
| User manual | 5.56  (1.12) | 5.8  (0.80) | 5.1  (1.37) | 5.54  (1.28) | 5.7  (1.05) | 5.2  (1.45) | 5.63  (1.19) | 5.7  (1.06) | 5.6  (1.36) |
| Pre-planning of surgery | 5.80  (0.98) | 5.7  (1.1) | 6  (0.63) | 5.53  (1.26) | 5.7  (1.19) | 5.2 (1.33) | 5.93  (0.77) | 5.9 (0.83) | 6  (0.63) |
| Fixation of patient marker | 6.00  (0.97) | 6.1  (0.54) | 5.8  (1.47) | 6.40  (0.61) | 6.3  (0.64) | 6.6  (0.49) | -a | -a | -a |
| Scan | 6.00  (0.63) | 5.9  (0.7) | 6.2  (0.4) | 5.60  (0.95) | 5.5  (0.92) | 5.8  (0.98) | 5.93  (0.77) | 5.8  (0.87) | 6.2  (0.4) |
| Registration 1  (Segmentation and labeling) | 5.73  (1.18) | 5.4  (1.28) | 6.4  (0.49) | 5.07  (1.53) | 4.7 (1.42) | 5.8  (1.47) | 6.00  (0.82) | 5.8  (0.87) | 6.4  (0.49) |
| Registration 2  (ROI setting and image matching) | 4.73  (1.57) | 4.8  (1.54) | 4.6  (1.62) | 4.47  (1.63) | 4.4  (1.62) | 4.6  (1.62) | 5.13  (1.50) | 5.1  (1.51) | 5.2  (1.47) |
| Verification | 5.60  (1.14) | 5.6  (1.02) | 5.6  (1.36) | 5.40  (1.31) | 5.5 (1.2) | 5.2  (1.47) | 5.73  (1.00) | 5.8  (0.75) | 5.6  (1.36) |
| Revision of surgical planning | 5.80  (0.83) | 5.9  (0.7) | 5.6  (1.02) | 5.93  (0.68) | 6 (0.63) | 5.8  (0.75) | 5.93  (1.29) | 6.3  (0.78) | 5.2  (1.72) |
| Navigation | 5.67  (1.07) | 5.9  (0.7) | 5.2  (1.47) | 5.93  (0.77) | 5.8 (0.75) | 6.2  (0.75) | 5.33  (1.45) | 5.2  (1.6) | 5.6  (1.02) |

a - : Fixation of Patient markers was not surveyed because they do not have any on-screen information.

# **Table S2.** ASQ result in nurses.

|  | Ease of completing the task Mean(SD) | | | Amount of time it took Mean(SD) | | | | Support information satisfaction Mean(SD) | | | |
| --- | --- | --- | --- | --- | --- | --- | --- | --- | --- | --- | --- |
|  | ALL | Less than 3 years | More than 3 years | | ALL | Less than 3 years | More than 3 years | | ALL | Less than 3 years | More than 3 years |
| User manual | 6.00  (1.23) | 6.15  (1.00) | 5.58  (1.51) | | 5.80  (1.63) | 5.86  (1.58) | 5.62  (1.50) | | 5.97  (1.23) | 6  (1.19) | 5.88  (1.15) |
| Preparations for use | 6.47  (0.62) | 6.45  (0.66) | 6.5  (0.5) | | 6.07  (1.12) | 5.91  (1.24) | 6.5  (0.5) | | 6.40  (1.02) | 6.36  (1.15) | 6.5  (0.5) |
| System operations | 6.27  (0.85) | 6.18  (0.94) | 6.5  (0.5) | | 6.20  (0.98) | 6.09  (1.08) | 6.5  (0.5) | | 6.2  (1.11) | 6.18  (1.19) | 6.25  (0.83) |
| Initialization manipulator | 6.13  (1.15) | 6.36  (0.88) | 5.5  (1.5) | | 5.87  (1.45) | 6.09  (1.16) | 5.25  (1.92) | | 5.87  (1.45) | 6.09  (0.67) | 5.25  (2.49) |
| Drape | 5.27  (1.84) | 5.64  (1.29) | 5.25  (2.49) | | 5.67  (1.62) | 5.82  (1.15) | 5.25  (2.49) | | 5.80  (1.51) | 6.09  (0.79) | 5  (2.45) |
| Preparation for surgery | 6.13  (0.88) | 6.18  (0.72) | 6  (1.22) | | 6.07  (0.93) | 6.09  (0.79) | 6  (1.22) | | 6.27  (0.93) | 6.36  (0.77) | 6  (1.22) |
| Scan | 6.07  (0.85) | 6.18  (0.83) | 5.75  (0.83) | | 5.87  (0.88) | 5.91  (0.90) | 5.75  (0.83) | | 6.07  (1.06) | 6.09  (1.08) | 6  (1.00) |
| Registration 1  (Segmentation and labeling) | 5.53  (1.50) | 5.82  (1.27) | 4.75  (1.79) | | 4.93  (1.81) | 5.09  (1.88) | 4.5  (1.5) | | 5.67  (1.85) | 5.82  (1.75) | 5.25  (2.05) |
| Registration 2  (ROI setting and image matching) | 4.47  (1.89) | 4.55  (1.88) | 4.25  (1.92) | | 4.40  (2.09) | 4.45  (2.15) | 4.25  (1.92) | | 5.13  (1.89) | 5.27  (1.71) | 4.75  (2.28) |
| Verification | 4.93  (1.77) | 5.18  (1.80) | 4.25  (1.48) | | 5.20  (1.38) | 5.27  (1.54) | 5  (0.71) | | 5.40  (1.74) | 5.55  (1.78) | 5  (1.58) |
| Navigation | 6.60  (0.61) | 6.64  (0.48) | 6.5  (0.87) | | 6.60  (0.61) | 6.64  (0.48) | 6.5  (0.87) | | 6.60  (0.61) | 6.64  (0.48) | 6.5  (0.87) |
| Use of the emergency stop switch | 6.73  (0.44) | 6.73  (0.45) | 6.75  (0.43) | | 6.60  (0.61) | 6.55  (0.66) | 6.75  (0.43) | | 6.73  (0.44) | 6.73  (0.45) | 6.75  (0.43) |
| Cleaning up after surgery | 6.47  (0.88) | 6.36  (0.98) | 6.75  (0.43) | | 6.40  (1.02) | 6.27  (1.14) | 6.75  (0.43) | | 6.40  (1.02) | 6.27  (1.14) | 6.75  (0.43) |

# **Table S3.** User interface improvements from usability tests.

|  | Before | After |
| --- | --- | --- |
| Scan |  | 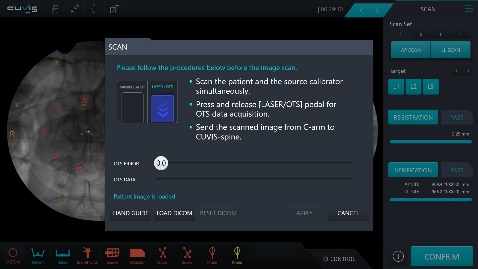 |
|  | Previously, OTS errors were displayed only as a graph, making it difficult to understand the exact number and determine how much error to consider, but by showing both numbers and graphs at the same time, it has been improved to help users make decisions when using the device. | |
| Registration |  | 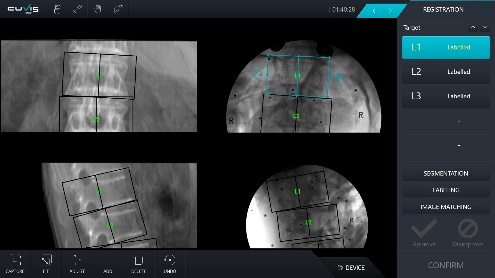 |
|  | When adjusting the ROI box, the rotation icon and the ROI box move icon, which were located at the top and bottom, were small and difficult to change, causing errors in usability test, time-consuming, and low satisfaction. We improved the user interface by moving the icons to the left and right for easier operation. | |
|  |  | 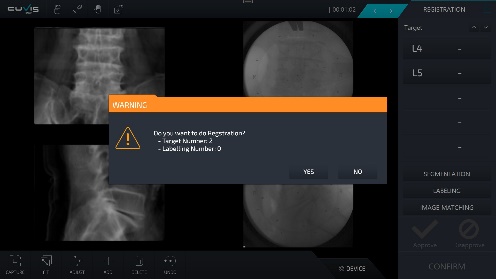 |
|  | After clicking the “image matching” button, there was no confirmation window to notify the user of the process, and it took about 3 minutes to match the image, and there were complaints about not being able to turn off or stop the window in the middle. To prevent such errors, we have improved the warning window to pop-up and confirm the target number and labeling before proceeding to the next step. | |
| Verification | 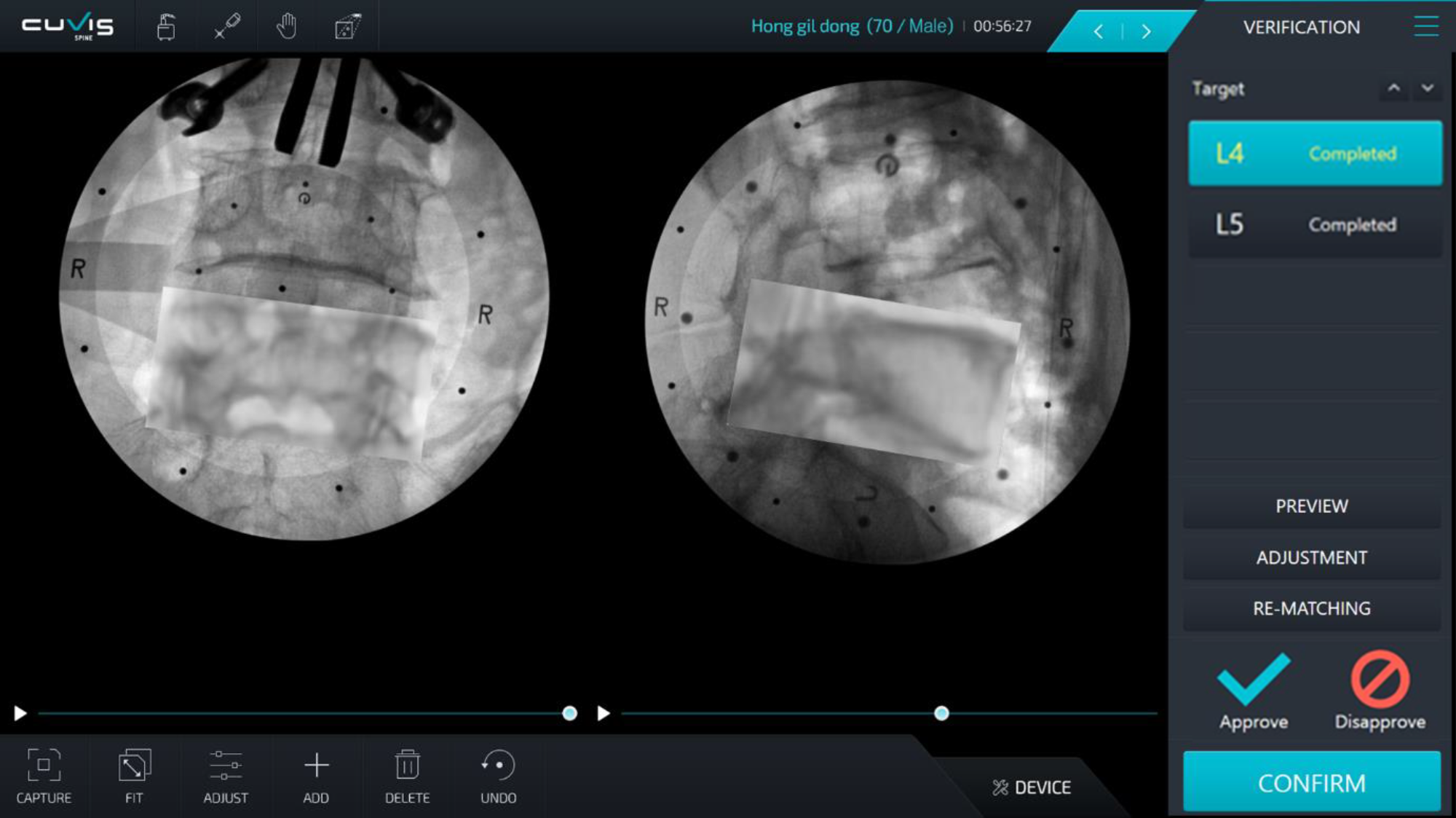 | 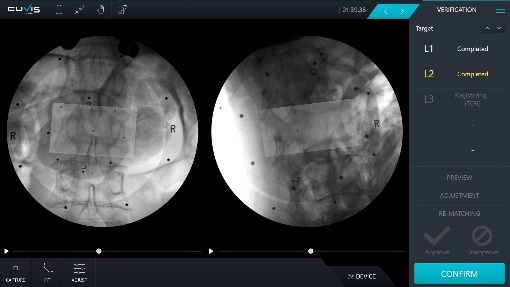 |
|  | When moving from registration to verification, the “Preview,” “Adjustment,” and “Re-matching” buttons were able to be clicked before the image was matched for each target, resulting in incorrect adjustment or rematching. To reduce errors, the user interface has been improved to disable the buttons until the image matching is completed. | |
| Revision of surgical planning | 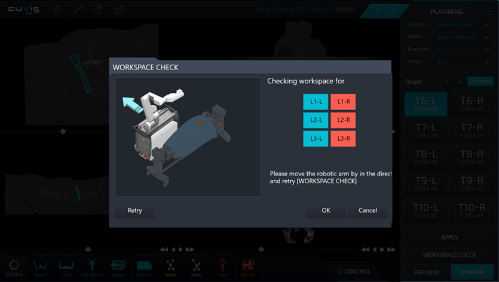 | 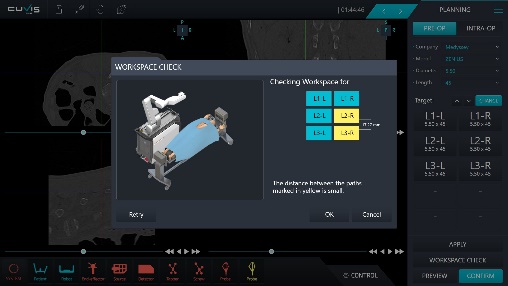 |
|  | When checking the workspace, users were able to see the change only through color, not being able to intuitively check the distance and causing usage errors and low satisfaction. To solve this problem, distance was displayed along with a color change to be able to intuitively check how much to move. | |
|  | 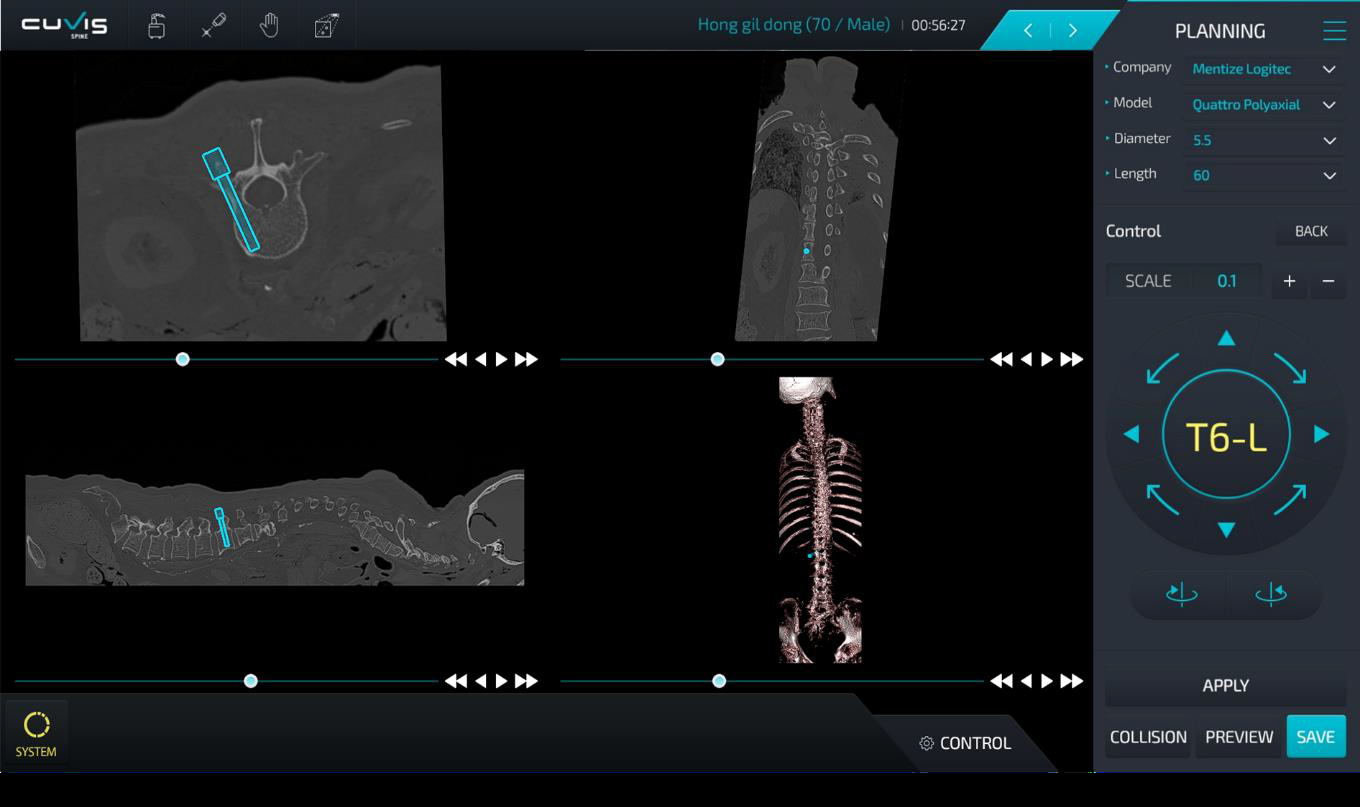 | 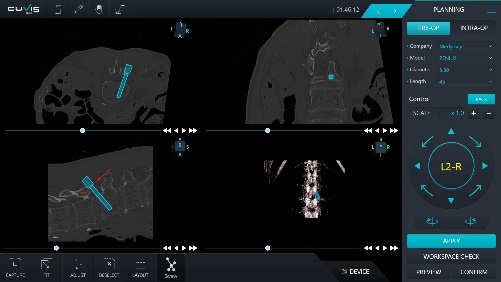 |
|  | When modifying the position of the screw, the “Save” button is highlighted, leading the users to press the “Save” button and not the “Apply” button to implement the changes. The user interface has been improved to highlight the “Apply” button so that the “Apply” button can be pressed first to apply the modified screw position. | |
